# SEM-EDX Study of the Degradation Process of Two Xenograft Materials Used in Sinus Lift Procedures

**DOI:** 10.3390/ma10050542

**Published:** 2017-05-17

**Authors:** María Piedad Ramírez Fernández, Sergio A. Gehrke, Carlos Pérez Albacete Martinez, Jose L. Calvo Guirado, Piedad N. de Aza

**Affiliations:** 1 Catedra Internacional de Investigación en Odontología, University Católica San Antonio de Murcia, Avda. Jerónimos, 135, 30107 Guadalupe, Murcia, Spain; mpramirez@ucam.ed (M.P.R.F.); cperezalbacete@ucam.edu (C.P.A.M.); jlcalvo@ucam.edu (J.L.C.G.); 2 Biotecnos Research Center, Rua Dr. Bonazo n° 57, 97015-001 Santa Maria (RS), Brazil; Sergio.gehrke@hotmail.com; 3 Instituto de Bioingenieria, Universidad Miguel Hernandez, Avda, Ferrocarril s/n, 03202-Elche Alicante, Spain

**Keywords:** hydroxyapatite, xenografts, tissue reaction, resorption, biocompatibility, biomedical applications

## Abstract

Some studies have demonstrated that in vivo degradation processes are influenced by the material’s physico-chemical properties. The present study compares two hydroxyapatites manufactured on an industrial scale, deproteinized at low and high temperatures, and how physico-chemical properties can influence the mineral degradation process of material performance in bone biopsies retrieved six months after maxillary sinus augmentation. Residual biomaterial particles were examined by field scanning electron microscopy (SEM) and energy dispersive X-ray spectroscopy (EDX) to determine the composition and degree of degradation of the bone graft substitute material. According to the EDX analysis, the Ca/P ratio significantly lowered in the residual biomaterial (1.08 ± 0.32) compared to the initial composition (2.22 ± 0.08) for the low-temperature sintered group, which also presented high porosity, low crystallinity, low density, a large surface area, poor stability, and a high resorption rate compared to the high-temperature sintered material. This demonstrates that variations in the physico-chemical properties of bone substitute material clearly influence the degradation process. Further studies are needed to determine whether the resorption of deproteinized bone particles proceeds slowly enough to allow sufficient time for bone maturation to occur.

## 1. Introduction

Alveolar bone resorption and pneumatization of the maxillary sinus after tooth extraction limit the quantity and quality of the bone needed for successful implant placement, especially in the edentulous posterior maxilla. Many authors have studied posterior maxillary atrophy rehabilitation, and have reported various maxillary sinus floor augmentation techniques that increase bone volume and height [1,2,3]. The maxillary sinus elevation procedure is currently considered the most predictable of pre-prosthetic surgical techniques. Maxillary sinus grafting, combined with Schneiderian membrane elevation, has been proposed to re-establish the ideal quantity and quality of bone prior to implant [4].

Grafting material is an important determinant of bone augmentation procedures being a success or a failure [5]. Ideal bone graft material should be biocompatible [6], increase bone volume in the grafted area to promote initial stability at implant sites [7], and be resorbed with time and be replaced with native bone [8]. The success of scaffold-based bone regeneration approaches strongly depends on the performance of the biomaterial used. Among the efforts made by regenerative medicine toward restitution ad integrum, scaffolds should be completely degraded within an adequate period of time. Degradation of bone grafts substitute materials involves both chemical dissolution (physico-chemical degradation) and resorption (cellular degradation by osteoclasts) [9]. Based on its physico-chemical properties, bone-graft material may be either resorbable or non-resorbable in relation to the extent of dissolution of Ca–P materials [10]. The factors that affect dissolution properties are similar to those that affect biodegradation or bioresorption [11].

Materials should also be fully degradable and this degradation should ideally match the osteogenic rate. However, no clear insight has yet been provided as to the exact relationship between the material properties of these calcium phosphate ceramics and their degradation behavior [12]. Therefore, long-term studies must be carried out to understand the pattern of biodegradation of xenografts and their influence on bone gain.

An ideal bone graft substitute has long since been sought for problems associated with gold-standard autologous bone grafts. Although autogenous bone is regarded as the gold standard, autogenous bone grafts can also exhibit unpredictable bone resorption, which may not be a desirable characteristic in sinus grafting. Indeed the practice of augmented sinuses using autogenous bone as the sole grafting material has been shown to undergo significant re-pneumatization and augmented volume loss according to several clinical reports found in the literature [13,14,15,16], which has led to searches for alternative graft materials. Therefore, the goal is to seek an ideal scaffold that provides good mechanical support temporarily while maintaining bioactivity, and which can biodegrade later at a tailorable rate [17]. Several grafting materials have been used for this treatment, but very few materials are presently considered useful [18]. Synthetic and natural calcium phosphate-based materials may be a suitable alternative for using autogenous graft [19]. Natural hydroxyapatite ceramics have drawn attention because it might be possible to use them as an alternative to autogenous free bone grafting due to their chemical composition and their biological and crystallographic similarity to the mineral portion of hard tissues [14]. Deproteinized xenografts, primarily constituted of natural apatites that are sintered or not, have good physico-chemical properties. However, information of manufacturers’ specifications sometimes contradicts clinical results. Some results have shown that the examined HA had variable physico-chemical properties, which disagrees with the manufacturers’ specifications. This discrepancy may affect the material’s performance [20]. Many reviews have discussed a number of biomaterials and their manufacturing processes for biodegradable scaffold fabrication, but very little work has been done to obtain biomaterials with patient-specific degradation rates [21,22,23,24].

One of the future challenges in bone tissue engineering is to design and manufacture biodegradable scaffolds with a homogeneous growth rate over their entire volume using pore size gradients or specific distributions. This requires manufacturing processes with higher resolution and biofabrication capabilities [25].

Bovine bone has practically unlimited availability and a striking physico-chemical and structural similarity to human bone [26]. However, some studies have pointed out that this material is not completely reabsorbable because it will completely disappear within one year. Moreover, the rate and mechanism of its reabsorption are still unclear [14]. Deproteinized porcine bone mineral has been recently developed and has become commercially available in maxillary sinus grafting, in which demineralized bovine bone mineral is widely used [27].

Xenografts are usually of bovine or porcine origin, and are constituted by HA similarly to human bone (Ca_10_ (PO_4_)_6_(OH)_2_). This type of graft can be deproteinized and/or demineralized under different physicochemical conditions, which selectively modulate the tissue response of the host organism [22].

Deproteinization is an indispensable process to eliminate antigenicity in xenograft bones. Valid strategies to eliminate the antigenicity of xenograft bones are of vital importance for the development of xenogenic bone graft substitutes [23]. Bone deproteinization methods have to allow the heterologous deproteinized bone to offer good biological safety and to meet all the demands of scaffold material for tissue engineering purposes [28]. Many research works on HA have centered on a wide range of powder processing techniques, and also on composition and experimental conditions, to determine the most viable synthesis method [29,30,31,32]. Some authors have reported the same observations in reactions to the microscopic structure in commercial products subjected to thermal deproteinization processes. Sintering temperature is considered an important factor that could alter the characteristics of HA [21,33,34,35,36]. However, the effect of sintering temperature on the physico-chemical properties of natural HA (HA of a natural source), especially HA from bovine bone, is not yet fully understood, and research in this area is still wide open [37]. The most important parameters that can affect the properties of HA are temperature and heat treatment duration [31].

Regarding the sintering characteristics of HA, the resulting microstructure and properties are not only influenced by the characteristics and impurities of materials, but are also found to depend on thermal history during the fabrication process [3].

Some studies have demonstrated that degradation processes are influenced by the material’s physico-chemical properties [26,38,39,40,41,42]. Different applications require materials with distinct resorption rates, which can be regulated by a mixture of several calcium phosphate phases [43]. Based on these data, the objective of this study was to compare the physico-chemical properties of two deproteinized HA materials, and to assess the influence of these properties on the degradation process of the material’s performance in retrieved bone biopsies following use in maxillary sinus augmentation.

## 2. Results

### 2.1. Characterization of Deproteinized Hydroxyapatite Materials 

**Figure 1:**
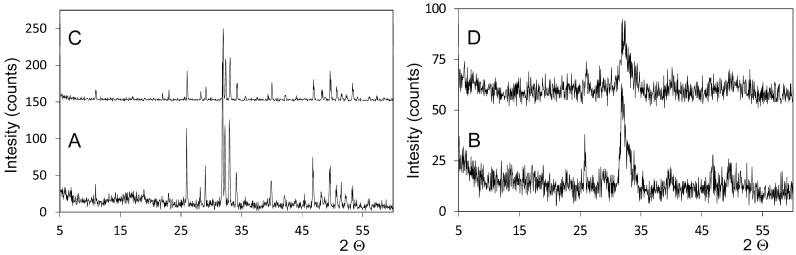
Laboratory XRD X-ray powder diffraction pattern of the obtained (**A**) DBHa and (**B**) DPHa materials, and also (**C**) the synthetic HA and (**D**) osseous matrix for comparison purposes.

Figure 1 shows the XRD pattern of the studied deproteinized hydroxyapatite materials, and the synthetic HA and osseous matrix for comparison purposes. As seen, the deproteinized bovine hydroxyapatite (DBHa) material presents a high crystalline HA with no other phase, which corresponds to high-temperature processing. Based on the XRD patterns, the crystal size of the DBHa xenograft implants is 732 nm. The deproteinized porcine hydroxyapatite (DPHa) material presents a low crystallinity HA phase (with a crystal size of 325 nm) with broad peaks related to low-temperature sintering 

**Figure 2:**
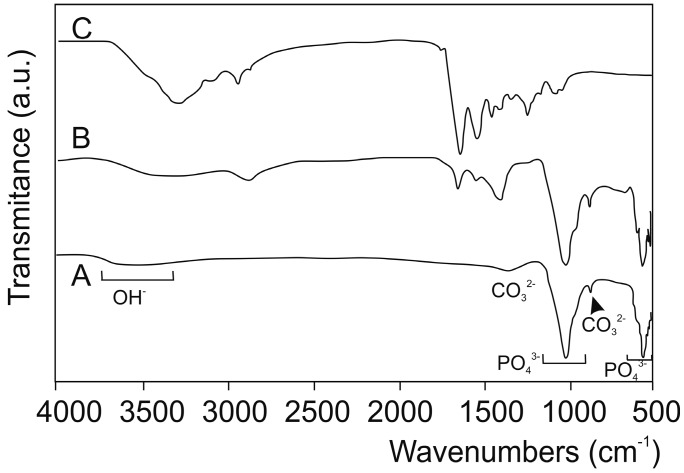
FTIR spectra of the obtained (**A**) DBHa and (**B**) DPHa materials and (**C**) collagen for comparison purposes.

Figure 2 includes the FTIR spectra of the obtained DBHa and DPHa materials, as well as the spectrum of collagen for comparison purposes. As expected, both the deproteinized HA materials show the typical bands brought about by HA, the main constituent of bovine and porcine bone: 1125–1040 cm^−1^ (ν3); 963 cm^−1^ (ν1), and between 550 and 610 cm^−1^ (ν4). These are the most intense phosphate stretching bands observed at around 1043 cm^−1^ and 1092 cm^−1^. In addition, a double band appears at 1410–1480 cm^−1^ (ν3) and a low-intensity band at 885 cm^−1^ (ν2), which correspond to the stretching vibrations of CO_3_^2−^, by substituting for phosphate in the apatite lattice that corresponds to natural carbonate-hydroxyapatite [44]. 

The third group of vibrational spectra includes the spectra related to the collagen present only in the DPHA material. The figure includes Type I collagen for comparison purposes. Above 1300 cm^−1^, almost all the bands are exclusively assigned to collagen vibrations, the exception being those brought about by CO_3_^2−^ at 1460 cm^−1^, and a broad band present at 3500, which is attributed to the presence of the structural OH- groups [12,45,46].

**Figure 3:**
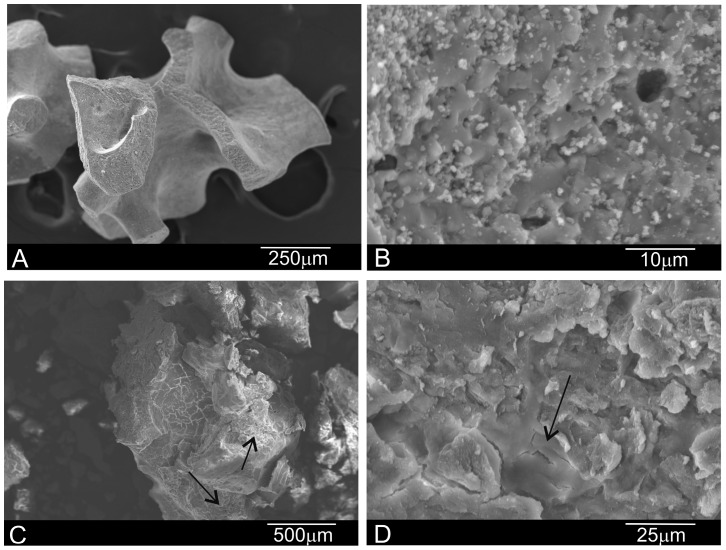
Scanning electron micrographs of the (**A**,**B**) DBHa and (**C**,**D**) DPHa xenograft materials before implantation [arrows = collagen].

Figure 3 shows the xenograft materials before insertions for maxillary sinus floor elevation. The SEM micrographs illustrate major differences depending on heat treatment. DBHa consists of 500–1000 μm average particles with rounded edges and 100 μm pores. At high magnification, the DBHa material shows a porous surface with white HA crystals (Figure 3A,B). DPHa has HA particles of about 600–1000 μm on average, with sharper edges and collagen (arrows in Figure 3B,C). Collagen is attached to the particle’s surfaces or by linking different particles. Figure 3D depicts details of a sample similar to the previous one, but shows a bundle of collagen over a smooth particle area.

EDX also denoted the differences between both xenograft materials (Table 1). The Ca/P ratio revealed a statistically significant difference in the EDX analysis of grafts before implantation.

Table 2 provides a comprehensive microstructural characterization of the xenograft materials and the synthetic HA and osseous matrix for comparison purposes. The tendency was for density to become higher with increasing heating temperature and lower porosity.

### 2.2. Results of Retrieved Bone Biopsies

The SEM-BSE cross-sections micrographs six months after the sinus augmentation of both materials are shown in Figure 3. It is important to highlight the absence of either inflammatory cells or fibrous connective tissue formation in the vicinity of the xenograft materials and around the newly formed woven bone, which would otherwise imply bone tissue intolerance to implants. The behavior of both implants differs. DBHa material is still present in the implantation area after six months, while the DPHa material is almost degraded.

**Figure 4:**
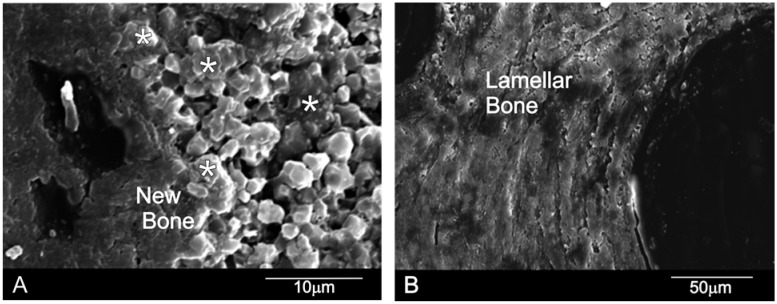
The SEM-BSE cross-sections of the (**A**) DBHa and (**B**) DPHa xenograft materials six months after sinus augmentation (* = residual graft particles).

Figure 4A confirms that the DBHa residual graft particles (*) were surrounded by newly formed bone, which presented mature bone characteristics, and the EDX analysis supported these findings (Figure 4). Six months after implantation in the DBHa group, most of the residual biomaterial had been resorbed. The patterns of resorption at the interface of the DBHa group show resorption regions mainly on its surface, and a more uneven surface morphology compared with DPHa. The newly formed bone is found in most of the convex augmented space. Newly formed bone is closely attached to the HA particles from the implant. For the DPHa xenograft (Figure 4B), behavior differs as an almost complete resorption zone is observed in them. The patterns of resorption at the interface of the DPHa group show numerous resorption regions, starting from inside the graft and moving toward its periphery. The DPHa group has the highest resorption rate compared with the other DBHa group.

**Figure 5:**
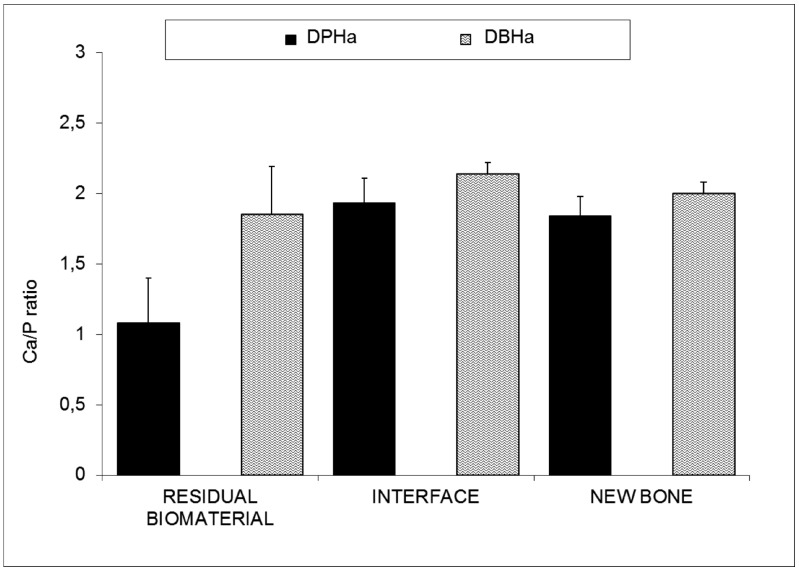
Comparisons between the DBHa and DPHa groups for each parameter (residual bone, interface, and new bone). The Kolmogorov–Smirnov test rejected normality for all the groups. Statistically significant differences were found for each parameter between the group comparisons (Mann–Whitney test, *p* < 0.0001).

Figure 5 shows the results of the Ca/P ratio of the retrieved bone biopsies following maxillary sinus augmentation. The EDX analysis indicated a significant decrease in the residual biomaterials (RB) compared with the initial composition (Table 1). The DPHa shows numerous regions of resorptions, with an average Ca/P rate of 1.08 ± 0.32 compared with the average Ca/P rate 1.85 ± 0.34 of DBHa. Statistically significant differences appear between groups at the interface and in new bone. The Ca/P ratio at the interface is 1.93 ± 0.18 for DPHa compared with 2.14 ± 0.08 of DBHa, and the ratio in new bone is 1.84 ± 0.14 for DPHa compared with 2.00 ± 0.08 of DBHa. In all cases, a drop in the Ca/P ratio percentage takes place in the residual biomaterial compared with the initial composition (Table 1), while the Ca/P ratio percentages gradually increase at the interface, which suggests an increase in the osteoinductive capacity of the materials and replacement with new bone on their periphery.

To investigate in detail the distribution of Ca/P in selected areas and individual biomaterial particles, line scans were carried out with a selection of different points from the implant through the middle to the periphery of samples to detect changes in the Ca/P ratios (see Figure 5), following the recommendation of Lindgren [47]. The EDX analysis of the residual graft material particles in the retrieved tissue revealed Ca/P to be in every variable relative proportion. The analysis indicated that this individual particle contained Ca/P, which was more concentrated in the interface area. This is consistent with the replacement of Ca/P with the precipitate as it gradually dissolves. 

**Figure 6:**
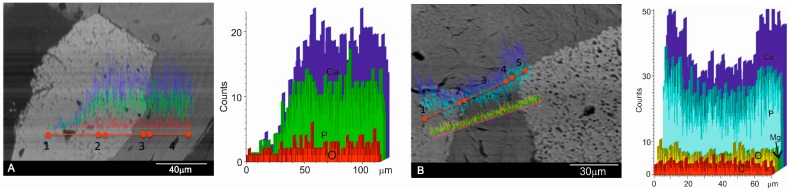
The SEM line-scan of the (**A**) DBHa and (**B**) DPHa xenograft materials six months after implantation showing the relative concentration of the principal ions along a line that passes through a graft biomaterial particle (point 1) and the interface (point 2) to the new bone (point 3) interface (point 4), and a graft biomaterial particle (point 5). In order to clarify, the scan results are also shown separately in the figure next to each SEM image.

The data of interest were found in the interface area, where the diffusion of ions was greater. According to the EDX analysis and the high magnification SEM examination of the interfaces made between both studied grafts and the surrounding tissue, the reaction zone was characterized by the intermittent presence of the calcium phosphate phase, which corresponded in structure and morphology to new bone tissue. The intermediate new bone contained calcium and phosphorous elements with average Ca/P ratios for DPHa 1.93 ± 0.18 and DBHa 2.14 ± 0.08. These results indicated bone chemical maturity, and reached the stoichiometric Ca/P ratio of natural bone. This compositional microcharacterization of the interface indicated that the calcium and phosphorous along the periphery of the implant interfaces remained well textured as the material’s degradation continued inside the implant. This led to increased porosity inside the material, which can be observed by comparing the SEM images of Figure 6. Faster degradation of DPHa compared to DBHa is observed, which is related with the material’s crystallinity (Figure 1).

**Figure 7:**
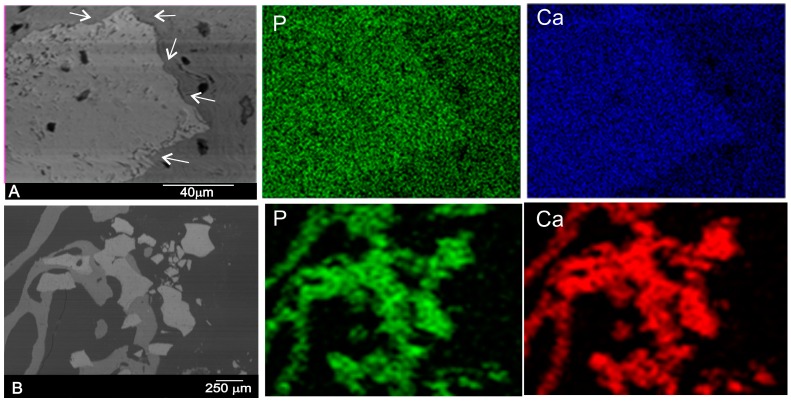
SEM image of the polished cross-section of a biopsy of the (**A**) DBHa and (**B**) DPHa xenograft material six months after implantation and the elemental X-ray maps of calcium and phosphorous (arrows refer to the irregular boundary with a partial degraded implant).

The SEM image of the polished cross-sections of implants six months after implantation is shown in Figure 7. The equivalent elemental X-ray maps of calcium and phosphorous are also presented. At six months after implantation, the outside DBHa implant surface (arrows in Figure 7A) presented active regions, where the degradation process of the material came about. These observations led to the conclusion that the interfacial activities at six months were already making full progress, which resulted in the remodeling of the interface in terms of its morphology and chemistry (calcium and phosphorous ions). The implant peripheral regions underwent intensive bone resorption, which produced significantly more irregular surfaces compared with the DPHa implant (Figure 7B). Figure 7B provides a biopsy of the DPHa-BSE image showing the resorbed DPHa graft in relation to the surrounding bone, a granular residual material consisting of areas with very few and smaller particles. These findings indicate the almost complete resorption of DPHa, and similar results in the middle and on the periphery of samples. 

According to the elemental X-ray maps and the SEM images of the interface between the DBHa and DPHa implants and natural bone, the reaction zone was composed of Ca and P phases, and was a short distance away from the reaction zone. No obvious morphological differences were observed between the newly formed bone and the old bone into which the implants were inserted.

## 3. Discussion

In the present study, the degradation process of two different xenogeneic bone substitute materials was evaluated in sinus floor elevation in a split mouth design. Both natural HAs differed significantly in processing temperature terms as DPHa was a porcine bone graft that was subjected to a low-heat (130 °C) chemical process to extract organic components, while DBHa was a sintered bovine bone graft that was subjected to high temperature (1200 °C). Conflicting results have been reported on the long-term fate of deproteinized bone. In the literature, the resorption of deproteinized bone has been a subject of controversy [9,22,37]. The mostly widely mentioned factors in the literature that presumably govern degradation are crystallography, stoichiometry, and porosity [48].

The manufacturing process, based on high-temperature heating, removes all organic components and proteins, and eliminates potential immunologic reactions. When deproteinization occurs at very high temperatures (1200 °C), it enhances material crystallinity, which is the case of DBHa [49].

In the present study, and according to the results of research on the effect of sintering temperature [31], our results reveal that sintering temperature is a critical factor that influences the phase stability, densification behavior, crystallinity, and porosity of hydroxyapatite ceramics. In line with other studies, we observed that changes in the composition and crystallinity of calcium phosphate-based materials from the manufacturer directly affect the clinical result [23] as biomaterials sintered at high temperature lead to non-absorbable products [9].

If we allowed biomaterials with the same initial particle size range (small size) to be used in both groups, 600–1000 μm for the DPHa group and 500–1000 μm for the DBHa, then particles might be degraded biologically or chemically.

The histological examination of 60 biopsies, taken from the 10 patients in this study, indicated statistically significant differences in the EDX analysis parameters between the DBHa and DPHa graft materials, and both before and after sinus augmentation. EDX provided information on the chemical elements present in the graft material and the surrounding tissues which can reveal changes in phase composition [50]. The purpose of this study was two-fold: to investigate the resorption of these grafts by examining the appearance of remaining particles by means of these techniques; to observe the interface between bone and biomaterial to determine whether any changes in the Ca/P ratio caused by component dissolution during the healing process could be detected in bone biopsies retrieved from maxillary sinus augmentation. The EDX analysis of the residual graft material particles in the retrieved bone biopsies six months after an augmented sinus lift revealed a Ca/P ratio in every variable relative proportion, which is useful for understanding the either resorbable or non-resorbable nature, and this classification is related to the dissolution extent of the Ca–P materials [10].

In the present study, the Ca/P variations in the graft material were analyzed in both grafts, and a decrease related to the dispersion of Ca and P through material degradation took place. An analysis was carried out with a selection of various points in order to take different points of interest from both the middle and periphery of samples to detect changes in the Ca/P ratios. The EDX analysis monitored the resorption process of both xenografts. An elemental analysis showed that a gradual diffusion of the Ca ions from the biomaterial occurred to the newly forming bone at the interface. Very few studies have used EDX analysis as a tool to understand the degradation process of a biomaterial [51].

According to the EDX analysis, the Ca/P ratio significantly lowered in the residual biomaterial (RB) compared with the initial composition in the DPHa group, and showed numerous regions of resorptions in relation to the DBHa group. However when we observed the DBHa group before and after, we observed a moderate decrease in the residual DBHa compared with the initial composition.

Changes in the composition and crystallinity of the calcium phosphate-based materials from one manufacturer to another, or even from different batches of the same manufacturer, directly affect the clinical result, and are a limiting factor for this material’s use [52].

Natural bone is made up of mainly fine carbonated HA crystals (65%) and collagen matrices (23%) with an organized three-dimensional geometrical structure, and has many excellent biological and mechanical properties [53].

Since industries created a self-setting calcium phosphate ceramic with low crystallinity HA, several commercial calcium phosphate ceramics have become available for clinical therapeutics in dentistry, such as biomimetic bone materials that contain HA/collagen, with high biocompatibility and similar characteristics to natural bone. This is the case of DPHa used herein. It is a xenograft that derives from cancellous-cortical porcine bone. The Tecnoss patented manufacturing process used to produce these materials achieves biocompatibility by avoiding temperatures above 130 °C, which would cause ceramization of granules by preserving part of the collagen matrix of the original animal bone. The result is a unique biomaterial that consists in mineral components and a very similar organic matrix to autogenous bone. According to the study of Kalkura [31], we observed the low crystallinity structure of the graft and the presence of two phases HA + Col by XRD and FTIR analyses in the previous characterization of the porcine bone mineral.

Crystallite size is an indication of the material’s crystallinity, which can be defined as the average size of a domain within a material that has a coherently diffracting monocrystalline structure. By increasing sintering temperature, the XRD patterns of the deproteinized graft materials exhibited an increased peak height and reduced peak width, which thus indicates increased crystallinity and a bigger crystallite size. Crystal size is inversely proportional to peak width. Broadening of peaks became evident at lower sintering temperatures, which indicates the initial crystal formation state. At higher sintering temperatures, crystal growth was evidenced by a sharpening to the peaks. For DPHa, the diffractogram exhibits broad peaks with a low signal-to-noise ratio, which correspond to a low-crystallinity material. The sharp, well-resolved peaks found in the XRD spectrum of DBHa indicate highly crystalline HA. So the DPHa sintered at low temperature had a less crystalline structure compared to the DBHa sintered at high temperature, and could also be more prone to degradation. Regarding physical properties, density tended to increase with an increasing annealing temperature.

Crystallinity is highly dependent on sintering temperature: the higher the sintering temperature, the more perfect the crystal and, thus, the lower the degradation rate. Resorption increases with decreasing crystallinity. Resorbable calcium phosphate materials are usually unsintered Ca–P materials [54].

Due to the low processing temperature, this material is claimed to preserve the structure and composition of natural bone components and enhances low crystallinity. Tissues respond differently to biomaterials with different crystallinities. The major differences in the adhesive response of cells to different crystallographic structures have been reported [55]. It has been shown that addition of collagen to HA results in higher bone remodeling activity compared to pure HA. According to another study, it would appear that addition of collagen to an HA graft could enhance phagocytotic processes [56].

Nannmark and Sennerby observed relevant significant levels of resorption of collagenized porcine bone particles [57]. According to these authors, this suggests that presence of collagen induces osteoclast adhesion to the biomaterial’s surface. Perhaps presence of collagen plays a key role in initiating resorption. Moreover, the low levels of residual graft material observed in our study confirmed a substantial resorption of the grafted material after six months, which is an added advantage for some authors. The elemental mapping of the residual porcine biomaterial at different points showed some categories of particles with different mean Ca/P ratios according to size. Compared with the original material composition, significant differences appeared before and after using DPHs. These findings marked the different resorption process stages. However, other research works [58] have used transmission electron microscopy of human biopsies following maxillary sinus elevation with porcine bone to show a few signs of resorption after five months. Their histological results indicated only the initial resorption of the biomaterial. Absence of collagen in the porcine bone used in Orsini’s study could explain its incomplete resorption and would, therefore, support our hypothesis. In any case, the rapid resorption of porcine bone that contains collagen would appear to claim an advantage for this material over other biomaterials that do not contain collagen. Other authors have described this characteristic in studies into HA. When a bone porcine paste, composed of 80% granulated mix and 20% pure collagen [59], was examined at three months, complete resorption and substitution with trabecular bone tissue were observed. However in our study, this rapid resorption was not an advantage as DPHa did not maintain the space and stability in the augmented area of the maxillary sinus floor.

Regarding porosity, another factor that presumably governs degradation, and is mentioned frequently in the literature, and also in our characterization study, it is likely that the porcine bone mineral particles’ microstructure and delicate porous morphology enhance the degradation of this material [60,61]. 

Given the low resorption rate of inorganic bovine bone, it has been reported to undergo no, or limited, resorption [14,62,63,64]. The present study’s findings are consistent with several reports found in the literature [65,66,67] in which an SEM analysis revealed a close relationship between the newly formed bone matrix and the bovine xenograft particle surface. Although histological and histomorphometric findings have often corroborated the clinical success of bovine bone mineral, the published literature includes a few descriptions of its ultrastructural features. The purpose of our evaluation was to, therefore, add to clinicians’ knowledge of this biomaterial by gaining an understanding of the interactions that occur in close proximity to bovine bone mineral. The EDX bone tissue analysis showed presence of calcium and phosphorus, and indicated presence of mineralized bone tissue on the particle surface. The results demonstrated that Ca ions gradual diffused from the biomaterial to the newly forming bone at the interface. This observation suggests that the graft surface might provide an optimal stratum for bone tissue ingrowth [64]. Moreover, the residual graft material levels observed herein confirmed a substantial resorption of the DBHa biomaterial after six months. The elemental mapping of the residual DBHa sintered biomaterial at different points showed some categories of particles with different mean Ca/P ratios according to size. Compared with the original material composition, a significant resorption of biomaterials was seen to be underway. It became evident that these findings marked different resorption process stages. In agreement with our clinical findings, a low osteoclast count over time would explain the long-term (six months, three years, and seven years) persistence of bovine bone biomaterial [64]. Conflicting results have been reported on the long-term behavior of bovine xenografts. Early studies reported that bovine xenografts are non-resorbable [68]. Taylor´s study describes signs of resorption [69], whereas others report that they are lacking [62,65]. Traini´s study discovered bovine bone biomaterial remnants remaining for as long as nine years. A follow-up study of sinus floor augmentation with bovine xenografts observed the gradual diffusion of Ca ions from the bovine xenograft to the adjacent newly forming bone at the interface as part of the resorption process [67]. 

However, other [65] studies done with human biopsies following maxillary sinus elevation with bovine bone have reported few signs of resorption after six months, with histological results indicating only initial biomaterial resorption. In the current study, we found an incomplete bone graft resorption phenomenon, whereby the biomaterial was seen to partially decrease. In spite of this limited resorption capacity, we observed how intergrain boundaries widened, and how the superficial hydroxyapatite crystallites on the implant surface were partially dissociated. These findings became visible by an elemental analysis done at different points on the periphery of samples. In the present study, the sites of interest for a point analysis were randomly selected at the graft site, and it was interesting to determine whether any differences in the Ca/P ratio could be detected in peripheral or central regions, as this could indicate different resorption rates. For DBHa, resorption occurred mainly in peripheral regions, but the studied DBHa was not completely resorbable during the study period, according to another study. 

According to Moon´s study, which also compared two xenograft materials prepared by a low-temperature deproteinizing technique (Bio-Oss) or a high-temperature (Cerabone) one in the sinus cavity, a significantly greater volumetric loss of the initial graft size for the non-sintered material was observed, which also occurred in our study. Bio-Oss has a significantly larger surface area and a smaller crystallite size compared with Cerabone [70]. In contrast with Fienitz´s study, a sinterized material was compared to a non-sinterized bovine bone substitute material by a sinus augmentation procedure. Despite their histological analysis revealing a lower resorption of the non-sinterized bone substitute material, the differences found were not statistically significant [71].

In line with our study, in a randomized controlled clinical trial, Lee et al. compared sinuses grafted with DPBM (deproteinized porcine bone mineral) and DBBM (deproteinized bovine bone mineral). These authors observed smaller sized residual biomaterials in the histological samples from the DPBM sites compared to DBBM sites, and despite using the same sizes for both biomaterials [27].

This might have a crucial impact on the resorption rate [43]. Hence, we conclude that the results of resorption in deproteinized biomaterials varied according to the manufactured process (sintered or not sintered). 

After implantation, biodegradation is critical as this allows for a space to be formed into which bone and vascular tissues can grow. Biodegradation can be envisioned as an in vivo process by which a material breaks down into simpler components, which reduces the complexity of chemical compounds by the action of cells, by simple physical breakdown and/or by chemical erosion [8].

A completely resorbable ceramic has been the goal of several studies. However, a high rate of resorption or solubilization can interfere with bone formation as the biomaterial may degrade faster than the rate of bone formation. These phenomena lead to a change in the bioceramic physical structure, which interferes with cell attachment [72]. Moreover, the release of high concentrations of calcium to the microenvironment changes pH, promotes a mild inflammatory response, and favors fibrous tissue formation [73]. Higher calcium ion levels have been shown to effect osteoclastic activity by varying from its inhibition to its stimulation, or there simply being no effects [74]. Instead presence of moderate extracellular Ca^2+^ that results from resorption activity might be involved in the stimulation of osteoblasts. Yamaguchi et al. showed that moderately high extracellular Ca^2+^ is a chemotactic and proliferating signal for osteoblasts, and its stimulates pre-osteoblast differentiation [41].

As DPHa material is the fastest to undergo remodeling, it can be considered the most active in resorption terms, but not in new bone formation. DBHa seemed to be a gradually resorbed material, and was partially substituted for newly formed bone according to our results. This increase in ions could possibly create areas of biological apatite on agglutinated Ca and P deposits and crystals which, in turn, would facilitate osteoconduction [63]. 

Some materials, such as autogenous bone, cannot withstand sinus pressure during the first several weeks and loses density and height over time [16]. An ideal bone graft material should be biocompatible, increase bone volume in the grafted area to promote initial stability at the implant sites, and be resorbed with time and replaced with native bone. In this study, DPHa was reabsorbed so quickly that it was unable to withstand sinus pressure. A repneumatization of sinus, or reduced augmentation—a phenomenon known as ‘slumping’—was noted [75]. 

However, the bone resorption process and the distribution of osteoclasts after deproteinized bone implantation have not been examined in detail, which are relevant for enhancing our understanding of the complex role of osteoclasts in bone tissue engineering [74]. 

An in vitro study with osteoclastic precursor cells in bone substitute materials has shown that there are specific parameters that inhibit or enhance resorption. Moreover, analyses of the bone–material interface have revealed that biomaterials composition significantly influences their degradation when they come into contact with osteoclasts. The crystallinity, grain size, surface bioactivity, and density of the surface seem to have a less significant effect on osteoclastic activity. In addition, the topography of the scaffold surface can be tailored to affect the development and spread of osteoclast cells [75].

This suggests that resorption of collagen deproteinized bone material occurs more quickly than bone formation. Collagen is absorbed mostly by enzymatic digestion. It is noteworthy that TRAP-positive osteoclastic cells have been seen on the surface of deproteinized collagen. This implies that collagen fibrils are resorbed not only enzymatically, but also phagocytically. This was the case of the non-sintered DPHa [76]. Our study showed that most of the newly formed bone in the augmented sinus spaces grafted with DPHa had disappeared six months after implantation, which caused the repneumatization of the sinus.

The study of Galindo et al. (2012) found that the osteoclast count significantly lowered, and a true reduction in biodegradation [77]. Further long-term studies are needed to determine whether resorption of deproteinized bone particles proceeds slowly enough to provide sufficient time for bone maturation to take place. Our results demonstrate that slowly resorbed sintered DBHa particles promoted stable maxillary sinus floor augmentation and inhibited the resorption of newly formed bone, maintained the space, and stably augmented the maxillary sinus floor. The newly formed bone showed minimal osteoclastic resorption. The slow biodegradation of DBHa bone particles tented the sinus lining, maintained the space, and stably augmented the maxillary sinus floor. Therefore, we conclude that DBHa bone particles can be successfully used as a bone graft material for sinus lift procedures.

The grafting material is an important determinant of bone augmentation procedures being a success or a failure. With the various bone grafting options available to surgeons, one must carefully match the clinical problem and the graft material’s capabilities. The sintering process provides strength to a finished material. However, when applied to bone, one of the most important factors is the possibility of materials being resorbed and substituted for newly formed bone.

This study assumed the difference in the physico-chemical properties between the influence of both grafts on the biological response produced by a biomaterial, and significant differences were found in relation to the degradation process after six months of healing. According to others studies, phase composition [78], chemical composition [79], porosity [80], the dispersant concentration on the pore morphology [58], particle size [81], the ultrastructural geometry of particles [82], surface roughness [83], and crystallinity [17], among others, are likely to affect ceramic solubility. Different applications require materials with distinct resorption rates [9], which may be adjusted for the desired purpose [84].

## 4. Materials and Methods

### 4.1. Deprotenized Hydroxyapatite Materials

The raw materials employed in this study were two different types of commercial deprotenized HA materials used in dentistry: deproteinized porcine hydroxyapatite (DPHa) processed at 130 °C called OsteoBiol^®^, deproteinized bovine hydroxyapatite (DBHa) processed in two steps: pyrolysis at 900 °C following ceramization at 1200 °C called Endobon^®^.

The mineralogical characterization of the powder materials was performed by X-ray diffractometry (XRD). XRD patterns were obtained in a Bruker-AXS D8Advance (Karksruhe, Germany) automated diffractometer and were compared with the database provided by the Joint Committee on Powder Diffraction Standards (JCPD). The following Scherrer formula was used to determine the crystal size of each material based on the relevant XRD pattern. Instrument broadening corrections were taken into account, and it was assumed that the lattice strain was negligibly small.

Dhkl=kλB12cosθhkl

In this equation *k* is the Scherrer constant (0.89), which depends on crystal shape, the diffraction line indexes [85] and the dispersion of the crystallite sizes of the powder [86]; is the wavelength of Cu K_α1_ (λ = 1.54056 Å); B_1/2_ corresponds to full width at half maximum (rad) for (hkl) reflection; and θ*_hkl_* is the diffraction angle (°). The line broadening of the (300) reflection that corresponds to the maximum intensity peak was used to evaluate crystal size.

The particles´ real density (sample mass/volume of the solid (excluding empty spaces) was determined by helium-gas pycnometry (Quantachrome Instruments, Boyton Beach, FL, USA) and the particles´ apparent density was determined by mercury porosimetry. Particles´ real density was determined after excluding sample interstices and most pores since the small volume of the gas molecules (He) enables their penetration in almost all the empty spaces. One exception was samples’ closed pores, i.e., those pores that do not open out to the surface. Density, measured in this way, provides the closest value to the sample’s solid density, which justifies the use of the term ‘real’ or ‘true’ density.

Microstructural examinations were done under a scanning electron microscope (SEM, HITACHI S-3500N, Ibaraki, Japan). Quantitative analyses were run by an Energy Dispersive X-ray Spectroscopy (EDX) system, coupled to the above-described electron microscope. Calibration was carried out with Bayer standards. A weight percentage was calculated from the measured net intensities with a program that corrects for the influences of atomic number, absortion, and fluorescence (ZAF corrections). Relative counting error εn was calculated as εn = (N^1/2^/N)·100 (N = accumulated counts) with a probability of 98.5%.

Fourier transform infrared spectroscopy (FTIR-ThermoNicolet IR200, Waltham, MA, USA) was used to provide information on the chemical composition and major functional groups. FTIR spectra were recorded between 400 and 4000 cm^−1^ at 2 cm^−1^ resolution. Pellets were prepared by mixing each sample powder with the KBr matrix at a level of 1 wt %. Background data were collected for the KBr matrix and subtracted from each spectrum. All the spectra were recorded at ambient temperature. Analytical-grade collagen samples (purchased from Sigma-Aldrich, Darmstadt, Germany) were also used for comparison purposes.

### 4.2. Surgery and Bone Retrieval

Ten partially edentulous patients (six women and four men), whose ages ranged from 41 to 71 years, came to the Department of Oral and Maxillofacial Surgery, who had <5 mm subantral alveolar bone in the vertical direction, determined by conventional tomographic radiography. They were treated by a maxillary sinus floor augmentation procedure and delayed implant placement. Patients who received treatment with bisphosphonates or steroids, those currently undergoing chemotherapy and/or radiation to the oral cavity, and those with uncontrolled systemic disease or endocrine disorders were excluded from the study.

The protocol for harvesting bone samples was approved by the University Ethics Committee and informed consent was obtained from all patients. This study is consistent with the ethical principles set out in the Declaration of Helsinki for experimentation on human subjects. All the patients received antibiotics prior to surgery. Each subject was required to take 2 g of amoxycillin 1 h prior to surgery. For any patient allergic to amoxycillin, 600 mg of clindamycin were administered 1 h before surgery. All the surgical procedures were performed under local anesthesia (lidocaine hydrochloride 2% with 1:100,000 epinephrine or mepivacaine/carbocaine 3%, without epinephrine). Maxillary sinus augmentation was performed as previously described Tatum [79]. The grafts material was carefully packed in the space created by mucous membrane elevation. DBHa was placed in a subantral compartment and DPHa was placed in the contralateral subantral compartment. Upon implant surgery, and six months after the healing period, a biopsy was taken for histology purposes at the time of implant placement. Bone cores were harvested using a 3 × 10 mm diameter trephine bur under sterile saline solution irrigation conditions. Sixty bone samples were retrieved for the analysis. Two biopsies per individual were obtained. Nevertheless, the biopsy value we used in our analysis was unique for each individual and was computed as the average value of its biopsies. 

### 4.3. Specimen Preparation and Analysis

In order to investigate the relationship between xenograft biomaterial particles and bone, cross-sections of the non-decalcified tissue were examined for an ultrastructural study in SEM-EDX. Human specimens were fixed by immersion in 4% formalin solution, dehydrated in a graded ethanol series, and embedded in plastic resin (Technovit A 7210VCL; Kulzer & Co., Hanau, Germany). Then specimens were polished by a manual grinder with 800 grit silicon carbide paper, mounted on an aluminum stub, and carbon-coated in an argon atmosphere with a sputtering machine (Polaron K550X Sputter Coater, Laughton, UK) for the SEM-EDX analyses. Back-scattered SEM imaging (BSE) was also employed to highlight the contrast among the resin, bone, and biomaterials; generally, resin appeared black, bone was gray, and the biomaterial had a brighter contrast than bone. In addition, the chemistry of the interphases and the chemical degradation process of the xenograft biomaterials were analyzed using X-ray elemental maps in the scanning mode. An analysis was carried out by selecting different points, and by taking distinct points of interest from both the middle and the periphery of samples to detect changes in the Ca/P ratios. Data were then collected from deliberately targeted sites of interest from within the residual biomaterial, which were close to and distal from new bone, and also at the bone-implant interface if present (on average, approximately 15 points/section, depending on biomaterial content). Additional information was obtained from line scans and elemental maps. More than 900-point analyses were carried out on the 60 biopsies. 

### 4.4. Statistical Analysis

The statistical analysis was performed by the computerized statistical package software (MedCalc v15.8, Ostend, Belgium). Comparisons between the DBHa and DPHa groups for each parameter (RB, INT, and NB) were made by the Student´s *t* (parametric data) or Mann–Whitney (non-parametric data) tests. 

## 5. Conclusions

The fastest resorption rate of the material was in DPHa group and was related to physico-chemical characteristic of this xenograft material. A significant difference in resorption time and in the stability of the material was found in DBHa, which showed greater stability and less resorption than the DBHa groups. The HA of porcine origin non-sintered with high porosity, low cristallinity, low density, high surface area, and low calcium/phosphate ratio presents low stability and high resorption rate. 

This study demonstrates that variations in the physical properties of a bone substitute material clearly influence in the degradation process, as a consequence, biomaterials can be designed to demand depending on the needs of resorption, dimensional stability, and handling needed for each case. Further studies are required to establish to what extent the acceleration or slow-down of resorption phenomena affects the capacity of the bone augmentation area to receive and integrate dental implants. Our results demonstrate that slowly resorbed deproteinized bovine bone particles promote the stable augmentation of the maxillary sinus floor and inhibit the resorption of the newly formed bone.

This study demonstrates the effect of sintering temperature on the physico-chemical properties of natural HA and the influence on the degradation process. A significant difference in the resorption time and the stability of the material was found in both groups. The DPHa non-sintered HA with high porosity, low crystallinity, low density, large surface area, and a low calcium/phosphate ratio presented a high resorption rate, but could not withstand sinus pressure. This led to the repneumatization of the sinus. The DBHa sintered HA showed low porosity, high crystallinity, high density, small surface area, and a high calcium/phosphate ratio, and presented a slow resorption rate that inhibited the resorption of the newly formed bone, tented the sinus lining, maintained the space, and stabilized an augmented maxillary sinus floor. 

## Figures and Tables

**Table 1:** Ca/P ratios of grafts before implantation. Values as medians. A statistically significant difference was found between DBHa and DPHa (Mann–Whitney test, *p* < 0.0051).

Ca/P Ratios (wt %)	DPHa	DBHa
**Mean**	2.22	2.31
**SD**	0.08	0.09
**Median**	2.22	2.28

**Table 2:** The physical characteristics of the two studied xenograft materials and the HA synthetic and osseous matrix for comparison purposes [HA = hydroxyapatite; Coll = collagen].

Materials Characterized	Real Density (g/cc)	Apparent Density (g/cc)	Surface Area (m^2^/g)	Total Porosity (%) ^a^	Phase/s	Particle Size (μm)	Crystal Size (nm)
DPHa	2.85	1.14	9.78	59.90	HA + Coll	600–1000	325
DBHa	2.98	1.51	2.77	49.13	HA	500–1000	732
HA Synthetic	3.16	1.62	3.10	46.4	HA	600–1000	731
Osseous Matri	1.46	1.24	–	69.3	HA + Coll	400–700	68.4

^a^ Corresponding to 1 µm < pores < 300 µm.
